# Response: “Commentary: Utility-free heuristic models of two-option choice can mimic predictions of utility-stage models under many conditions”

**DOI:** 10.3389/fnins.2015.00299

**Published:** 2015-08-28

**Authors:** Steven T. Piantadosi, Benjamin Y. Hayden

**Affiliations:** ^1^Department of Brain and Cognitive Sciences, University of RochesterRochester, NY, USA; ^2^Center for Visual Science, University of RochesterRochester, NY, USA

**Keywords:** decision making, value comparison, heuristics, dimensional prioritization, value correlate, utility

In his accompanying commentary on our recent paper, Dr. Padoa-Schioppa identifies two putative errors in our manuscript (Piantadosi and Hayden, 2015). Both reflect basic misunderstandings of our arguments, as well as those of Tversky (1969), whose work ours is an extension of.

First, he argues that some commodities are inherently incommensurate, such as different juice flavors (“there is no parametric dimension along which two flavors can be assigned a scalar value”). This argument seems appealing because it is difficult to think of a single algorithmic function that would describe a juice flavor as a single number. So in a colloquial sense, juices could indeed by called incommensurate.

However, most models of choice assume, either tacitly or explicitly, that there is an intermediate stage during which each dimension is represented in a scalar manner and may there be deformed. A famous example is prospect theory (Kahneman and Tversky, 1979). In PT, gains are transformed using a convex utility curve and probability is deformed by a different curve; these transformed scalars are then combined into a single utility variable (Figure 1A). The same concept can be extended to thinking about very abstract goods like juice flavor, the funniness of a joke, or the intellectual appeal of a novel theory (Figure 1B). To do so, one may use a lookup table, as illustrated in our example. One example of a model that involves such a lookup table is Padoa-Schioppa and Assad (2006). In that paper, the authors propose that juices can be given a scalar value parameter, and that this parameter can be experimentally observed through preferences.

**Figure 1:**
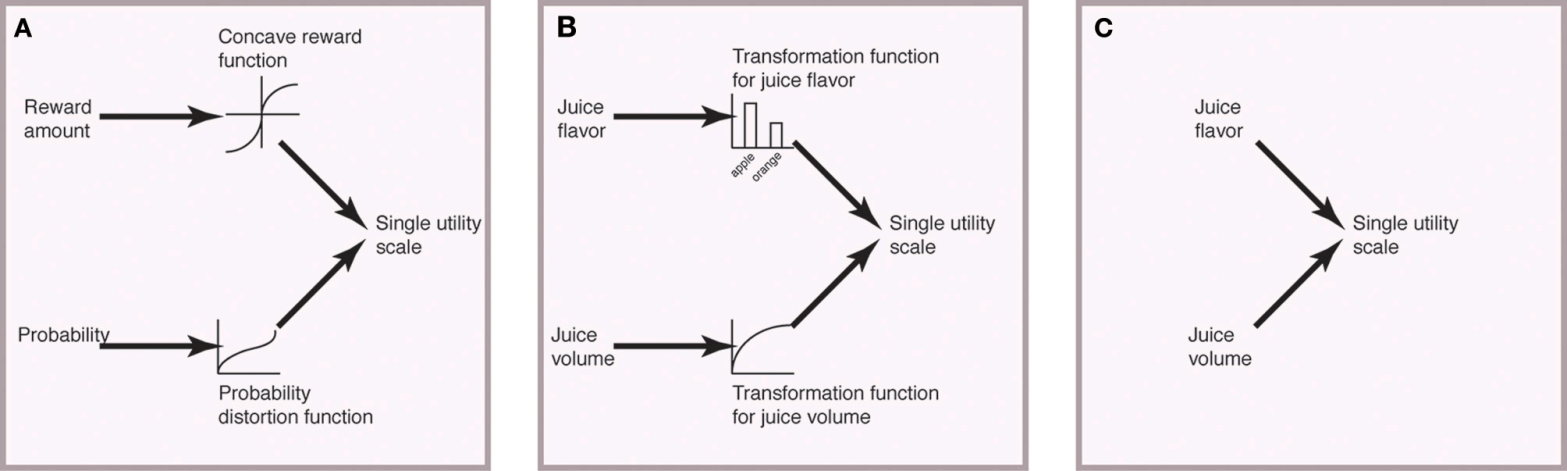
**Illustration of simplified process models for choice**. **(A)** In Prospect Theory (Kahneman and Tversky, 1979), reward amount and probability are both transformed into a single scale, but the two scales are different dimensions. These are the combined into a single utility scale. (Some aspects of PT are ignored here). **(B)** Choices between dissimilar goods that differ in complex ways can be solved in an analogous manner. The abstract dimensions (here, juice flavor) can be transformed using a lookup table (illustrated here) or some other method, before combining into a single utility scale. **(C)** A truly incommensurate good is combined to create a utility signal without an intermediate stage.

These idea of an intermediate stage is a critical part of several famous economic models (Bernoulli, 1738; Tversky, 1969; Kahneman and Tversky, 1979), including Padoa-Schioppa's own work, and has a direct neural correlate in the orbitofrontal cortex (Blanchard et al., 2015). A different possibility would be that it is impossible to construct an *intermediate-stage* model for a given choice process (Figure 1C). In such cases our ideas do not apply, nor would Tversky's. Some models that do not have an intermediate stage include decision by sampling, elimination by aspects, and query theory, as well as other sampling-based heuristic approaches (Tversky, 1972; Stewart et al., 2006; Johnson et al., 2007; Hayden and Platt, 2009).

Second, Dr. Padoa-Schioppa is concerned that there are well-defined value comparisons to which our methods do not apply. We clearly acknowledged this point in our original manuscript. Our formalization provides a way to recognize utility functions it does apply to; this is a strength of our approach.

However, while the example he gives was not discussed in our manuscript, it is straightforwardly covered using the approach we advocate. Indeed, the set of contexts to which our arguments apply is somewhat larger than we stated in the original article. Specifically, it applies to any context in which the choice can be modeled by a utility equation and then that equation can be rearranged to avoid the utility stage.

Dr. Padoa-Schioppa discusses a choice between gambles with probability (P_1_) of reward (R_1_) and fixed costs (C_1_), where choice is determined by:

(1) $${\text{P}}_{1}\cdot {\text{R}}_{1}-{\text{C}}_{1}>{\text{P}}_{2}\cdot {\text{R}}_{2}-{\text{C}}_{2}.$$

Following a method much like the one we presented (that is, first computing relative differences and then rearranging the terms), this choice is equivalent to:

(2) $$\frac{\frac{p}{P}+\frac{r}{R}}{\frac{c}{C}}>\frac{C}{P\cdot R}$$

where the uppercase letters are the average value in each dimension and the lowercase are half the difference.

To translate into prose, the decision-maker does the essentially same thing as in the examples used in our paper: she computes a normalization term that depends on average values, and then computes the ratio of the dimension-free relative differences for gains, and asks whether that ratio is greater or less than the relative difference for costs.

Thus, this example thus does not challenge our arguments, but instead enhances them. Moreover, it endorses our bigger (and ultimately very simple) point: given the power and flexibility of algebra, it is often possible to create process models that have no utility stage but make identical predictions to ones that do through a simple rearrangement of terms.

## Conflict of interest statement

The authors declare that the research was conducted in the absence of any commercial or financial relationships that could be construed as a potential conflict of interest.
